# Bird on Urinary Deposits,—Frick on Renal Diseases

**Published:** 1852-05

**Authors:** 


*Bird on Urinary Deposits.—Frick on Renal Diseases.*

The chemico-physiological investigation of the urine has added much to our knowledge of pathology; we propose, therefore, to present in a condensed form some of the more practical conclusions arrived at in the works whose titles form the caption of this article.

It has been pretty well ascertained that the function of the kidneys is, primarily, to eliminate from the system the effete nitrogenized compounds; and, secondly, to act as emunctories for carbonaceous substances in case of the torpidity of other organs.

The solids of healthy urine, consisting of urea, uric acid, inorganic salts, coloring matter, and a few other substances, are derived mainly from the metamorphosed albuminous tissues. The amount of each of the solid constituents is subject to but slight variation, without indicating positively diseased action, while the quantity of water, the average being from 30 to 40 fluid ounces daily, may be largely increased by the quantity or quality of the fluids taken into the stomach, or by a suppression of the cutaneous exhalation, and still not be considered as abnormal; the amount of water may also be very much diminished by accidental circumstances, and still not be incompatible with health.

The specific gravity of urine varies during the day; that passed in the morning, before eating—the urine of the blood—being less than that passed after eating—the urine of the food; the average for the 24 hours being nearly 1.021. If the same amount of solids be excreted in the 24 hours, it is evident that the specific gravity will be increased by a deficiency, and decreased by an excess in the quantity of water. We cannot be too careful, therefore, in drawing inferences from the quantity of fluid or from its specific gravity alone. As a general rule, if the variation from the normal standard of the specific gravity be inversely as that of the quantity of water, disease is not indicated.

A suppression of the cutaneous exhalation is one of the most frequent causes producing change in the quantity and quality of the urine. The water of the blood, usually passed off from the surface, is turned inward and finds an exit from the system through the kidneys, carrying with it the solids, amounting to about 107 grs. daily, that should have been excreted by the skin, and producing in the urine an excess of the urate of ammonia which disappears as soon as the determination to the surface is restored.

A similar condition of the urine may be produced by taking into the stomach a large amount of indigestible food which, instead of being perfectly assimilated and passing off as healthy urine, is converted into uric acid and urate of ammonia.

The following extract from Dr. G. Bird, presents in a concise form the pathological indications of this diathesis:

"Whenever an excess of uric acid or its combination with bases occurs in the urine, a normal quantity of water being present (30 to 40 ounces in twenty-four hours), it may safely be inferred that one or other of the following states exist:

| | |
|---|---|
| A. Waste of tissue more rapid than the supply of nitrogenized nourishment, as in | Fever, acute inflammation, rheumatic inflammation, phthisis. |
| B. Supply of nitrogen in the food greater than is required for the reparation and supply of tissue, as in | Excessive indulgence in animal food, or the quantity of food remaining the same, with too little bodily exercise. |
| C. Supply of nitrogenized food not being in excess, but the digestive functions unable to assimilate it. | All the grades of dyspepsia. |

| | |
|---|---|
| D. The cutaneous outlet for nitrogenized excreta being obstructed, the kidneys are called upon to compensate for the deficient function. | All, or most stages of diseases, attended with arrest of perspiration. |
| E. Congestion of the kidneys, produced by local causes. | Blows, and strains of the loins, disease of genital apparatus. |

An excess of uric acid, or urates, may then exist without indicating any serious derangement requiring the aid of medicines; on the other hand it may point to a dangerous difficulty, requiring active interference; and this can only be determined by an attention to other symptoms, such, for instance, as the presence or absence of the general symptoms of inflammation; the quantity and quality of the food; the condition of the digestive organs; the state of the skin; and the existence or non-existence of the usual signs, indicating disease of the genital or urinary organs.

The treatment, in cases where any is necessary, must be determined by the pathological indications, remembering always that the abnormal condition of the urine is not *the disease*, but simply the *evidence* of disease.

Urate of ammonia is readily distinguished by its perfect solution on the application of heat; and, if the microscope is used, by its amorphous, granular appearance; and, on the addition of hydrochloric or nitric acid, by the formation of uric acid crystals.

Phosphoric acid in combination with earthy or alkaline bases, is one of the constituents of healthy urine. These salts are derived from food, and from the albuminous tissues, which, during the process of disintegration, yields them in large quantities. Their presence in normal proportions in healthy urine, gives rise to no deposit, but in disease they often accumulate so as to alter the sensible properties of the secretion. The following extracts are from Dr. Bird's work:

"Deposits of these salts are always white, unless colored with blood; soluble in dilute hydrochloric acid, and insoluble in ammonia or liquor potassæ. On heating the urine, the deposit undergoes no further change, except agglomerating into little masses. Mucus, pus, and blood, are often present in the urine, and mask the chemical characters of the deposit."

"The physical appearance presented by the deposits of the earthy phosphates varies extremely; sometimes, especially when the triple salt forms the chief portion of the deposit, it falls to the bottom of the vessel as a white crystalline gravel. If but a small quantity of this substance be present, it may readily escape detection by remaining for a long time diffused through the urine; after a few hours' repose, some of the crystals collect on the surface, forming an iridescent pellicle, reflecting colored bands like a soap-bubble or a thin layer of oil. If then, the lower layers of the urine be placed in a watch-glass, and held obliquely over the flame of a candle or any strong light, a series of glittering points will become visible from the reflection of light from the facets of the minute prisms of the salt.

"The phosphates will often subside towards the bottom of the containing vessel like a dense cloud of mucus, for which they are frequently mistaken. Not unfrequently they will, in very alkaline urine, form dense masses in the urine, hanging in ropes like the thickest, puriform mucus, from which it is utterly impossible to distinguish them by the naked eye. Their disappearance on the addition of hydrochloric acid will at once detect their true nature. Where, as frequently occurs, a large quantity of ropy mucus, pus, or blood, co-exist with the phosphates, no mode of investigation can be so satisfactory as the examination of a few drops of the urine between two plates of glass, by the microscope, when the characteristic crystals of the phosphates are readily recognized."

The pathological indications of the triple phosphates are given by Dr. Frick, in the following paragraph:

"When these salts exist in the urine as a deposit, they are usually indicative of serious disturbance, and often of organic disease, unless they have occurred as a result of decomposition. But to this there are exceptions; for instance, medical students, or those who breathe for any length of time an atmosphere charged with ammonia, are liable to suffer with a deposit of this salt in their urine. It has also been observed in convalescence from acute disease, and in some cases of indigestion. But with these exceptions, it is most often found associated with a calculus in the bladder, or some organic mischief of that organ or the kidneys. There is always co-existent with the presence of this salt in the urine, a depressed state of nervous energy, sometimes local only, but at others general. For instance, we find it frequently in persons who are old and infirm, or in those who have received some blow or strain over the loins. The general symptoms are those which are consequent upon depressed nervous energy, and almost all persons suffering with this condition of urine, are regarded as laboring under hypochondriasis, or severe dyspepsia."

The indications for treatment, as is evident from the above, are varied; the difficulty being sometimes local and sometimes constitutional. The use of acids, especially the organic, have a tendency to maintain an acid re-action of the urine and thus secure a complete solution of the earthy salts; but, as Dr. Bird remarks, "all that is effected by such treatment is to mask a symptom, and an important one, of the progress of the malady." Cases in which the phosphates form deposits in the urine are divided by Dr. B. into four classes, "the existence of one or other of which must be deduced from the symptoms presented by the patient:

"A. Cases in which dyspepsia, often to an aggravated extent, with some febrile and nervous irritation, exists independently of any evidence of antecedent injury to the spine.
"B. Cases characterized by high nervous irritability, with a varying amount of marasmus, following a blow or other violence inflicted on the spine, but without paralysis.
"C. Cases in which the phosphatic urine co-exists with paraplegia, the results of spinal lesion.
"D. Cases of diseased mucous membrane of the bladder."

The first and fourth of these classes are frequently met with, in the latter of which Dr. B. has found much benefit to result from acid injections, by which the bladder was freed from the phosphates incrusting it. The treatment, however, of each class must be directed by general principles, rather than by specific rules.

Oxalate of lime is a salt so frequently found in the urine, and so often associated with disease, of which it furnishes one important indication, that it requires some notice.

Dr. B. first called the attention of the profession to this deposit. In his work he discusses somewhat at length its origin, and concludes that, except in cases where it has been taken into the system,

"It can only be referred to the same source as the accompanying urea and extractive matters, (which are always in excess,) viz.: an exaggerated activity of the second stage of the secondary or destructive assimilation, the metamorphosis of tissue of Liebig."

On this hypothesis he accounts for the emaciation almost always present in this diathesis. Its detection requires the aid of the microscope. The therapeutical indications are, to correct any disordered condition of the digestive organs, and restore as far as possible the general health; to avoid all depressing influences; to use a carefully regulated diet, consisting of animal and vegetable food in about equal proportions; and, in addition, the persistent use of the nitro-muriatic acid, in the proportion of about two parts of the latter to one of the former, and eight parts of water; of this mixture from ten to twenty drops may be taken in sweetened water 3 or 4 times daily.

The dumb bells, which till recently have been considered oxalates of lime, are believed by Dr. Bird to be an oxalurate of that base, while Dr. Frick maintains that they contain no lime, but are modified uric acid crystals, perhaps undergoing chemical changes,—a proposition which, in our judgment, requires proof. Dr. B. has demonstrated by chemical analysis the existence in the dumb bells of lime, and the microscope of Dr. F. has not demonstrated its non-existence.

The organic substances of the most importance in the urine, are mucus, pus, blood, sugar, and albumen, the latter of which is interesting from its frequent connection with organic disease of the kidneys.

The detection of albumen is easy, being simply the application of heat and the addition of nitric acid, either of which alone should, if albumen be present, produce a white, flocculent deposit. Alcohol produces the same result.

During the last year, there have been admitted two cases into the Ill. Gen. Hospital, and one to the U. S. Marine Hospital, with the urine charged with albumen. In two of the cases fibrinous casts of the tubuli were found. One died, and two were discharged apparently cured. One of the cases discharged was reported in this Journal—the other was treated mainly with cod liver oil and phos. lime. He improved rapidly in strength and general appearance.

In conclusion, we are compelled to say in relation to Dr. F.'s book, that we think he aimed at too much or too little. The name, "On Renal Affections," is a misnomer. Diseases of the kidneys are only incidentally treated of, the entire work being devoted to the physiology and pathology of the urine.

It is needless to say any thing of the work of Dr. Bird. To those not already familiar with it, the name of its distinguished author is a sufficient guaranty for its practical value. J.

